# 

**Published:** 1897-01

**Authors:** 


					﻿CONTENTS.
ORIGINAL ARTICLES—
A Chemical Study of Nasal Syphilis. By Dunbar Ray, A. B.,
M. D., Atlanta, Ga..................................  1
Report of a Case of Croupous Dysentery. By G. O. Jones, M.
D., Lithonia, Ga....... :.......................... 11
SOCIETY REPORTS—
Southern Surgical and Gynecological Association...... 13
SELECTIONS AND ABSTRACTS—
The Use of Alcohol in Sudden Illness..................29
EDITORIAL.................................... 32
NOTES......................................   35
CORRESPONDENCE.............................   37
BOOK REVIEWS................................. 40
SPECIAL NOTES................................ 42
PRESCRIPTIONS............................     51
				

